# Bioelectrical impedance analysis as a nutritional assessment tool in Autosomal Dominant Polycystic Kidney Disease

**DOI:** 10.1371/journal.pone.0214912

**Published:** 2019-04-04

**Authors:** Hyunjin Ryu, Hayne Cho Park, Hyunsuk Kim, Jongho Heo, Eunjung Kang, Young-Hwan Hwang, Jeong Yeon Cho, Kyu-Beck Lee, Yun Kyu Oh, Kook-Hwan Oh, Curie Ahn

**Affiliations:** 1 Department of Internal Medicine, Seoul National University Hospital, Seoul, Korea; 2 Department of Internal Medicine, Hallym University Kangnam Sacred Heart Hospital, Seoul, Korea; 3 Department of Internal Medicine, Chuncheon Sacred Heart Hospital, Chuncheon, Korea; 4 National Assembly Futures Institute, Seoul, South Korea; 5 Truewords Dialysis Clinic, Incheon, Korea; 6 Department of Radiology, Seoul National University Hospital, Seoul, Korea; 7 Department of Internal Medicine, Kangbuk Samsung Hospital, College of Medicine, Sungkyunkwan University, Seoul, Korea; 8 Department of Internal Medicine, Seoul National University Boramae Medical Center, Seoul, Korea; 9 Department of Internal Medicine, Seoul National University College of Medicine, Seoul, Korea; University of Mississippi Medical Center, UNITED STATES

## Abstract

**Objective:**

Autosomal dominant polycystic kidney disease (ADPKD) patients with massive organomegaly suffer from pressure-related complications including malnutrition. In this study, we analyzed the efficacy of segmental bioelectrical impedance analysis (BIA) for objective and quantitative nutritional assessment in ADPKD patients

**Design and methods:**

We conducted a cross-sectional study, to evaluate the clinical utility of segmental BIA for assessing the nutritional status of ADPKD patients. BIA measurements was assessed according to modified subjective global assessment (SGA) scores and were compared with data from a healthy population. The association between BIA measurements and the height adjusted kidney and liver volumes (htTKLV), were analyzed.

**Subjects:**

A total of 288 ADPKD patients, aged ≥ 18 years old, were analyzed

**Main outcome measures:**

Nutritional status was evaluated with SGA and segmental BIA. The htTKLV were measured in each patients using computed tomonography images.

**Results:**

Higher ratios of extracellular water to total body water (ECW/TBW) in the whole-body (ECW/TBW_WB_), trunk (ECW/TBW_TR_), and lower extremities (ECW/TBW_LE_) and lower phase angle of lower extremities (PhA_LE_) correlated with lower SGA scores in the ADPKD population and in both gender. The four parameters, ECW/TBW_WB_, ECW/TBW_TR,_ and ECW/TBW_LE_ of >0.38 and PhA_LE_ of <5.8 θ were associated with malnutrition in ADPKD patients. These correlations were preserved in the subgroup analysis for chronic kidney disease stages 1-3A. Compared to healthy populations’ data, body fluid parameters and segmental ECW/TBW values, except for the upper extremities (ECW/TBW_UE_), were greater in ADPKD patients. Increased htTKLV was an independent risk factor for malnutrition in ADPKD. The highest correlation with htTKLV was observed for the ECW/TBW_TR_ (r = 0.466), followed by ECW/TBW_WB_ (r = 0.407), ECW/TBW_LE_ (r = 0.385), PhA_LE_ (r = -0.279), and PhA_TR_ (r = 0.215).

**Conclusions:**

These results demonstrated that segmental BIA parameters of ECW/TBW_WB_, ECW/TBW_TR_, ECW/TBW_LE_ and PhA_LE_ provide useful information on nutritional status including the impact of organomegaly in ADPKD.

## Introduction

Malnutrition in chronic kidney disease (CKD), also known as protein-energy wasting is one of the strongest predictors of mortality and morbidity [1, 2]. Not only anorexia or inadequate intake of nutrients due to uremic symptoms in CKD patients but also inflammatory conditions and oxidative stress increase malnutrition risk in CKD patients [3]. In previous studies, nutritional markers such as serum albumin, creatinine, body mass index (BMI), and subjective global assessment (SGA) score were independent predictors of death and treatment failure in CKD [4, 5]. Besides, the pre-transplant nutritional status in CKD patients are known to affect the outcomes of kidney transplantation [6, 7]. Therefore, efforts have been made to establish guidelines for proper nutritional assessment and intervention to improve the outcome of CKD patients [8].

In autosomal dominant polycystic kidney disease (ADPKD), renal function decreases and the kidney and/or liver volume increases as the disease progresses. In addition to decreased renal function, organomegaly due to cyst growth is another risk factor for malnutrition in ADPKD, causing pressure-related symptoms and complications [9, 10]. Therefore, regular assessment of nutritional status and timely intervention are important in ADPKD patients [11, 12].

Subjective global assessment (SGA) is a standard nutritional assessment tool that has been well-validated in CKD patients [13, 14]. However, the practical applications of SGA are limited, as it is a non-continuous and subjective scoring system that cannot detect subtle individual changes during regular follow-ups. Thus, other methods for monitoring nutritional status in ADPKD patients are required in addition to SGA.

Bioelectrical impedance analysis (BIA), which uses electrical currents and impedance to assess fluid status and body composition, could be an option for assessing nutritional status in ADPKD patients. Its use has been validated in various conditions, including CKD [15–18]. Increased ratio of extracellular water to total body water (ECW/TBW), change in body composition, and reduced phase angle (PhA) are known to be related to malnutrition [16, 19]. The main advantages of BIA over other methods (Dual-energy X-ray absorptiometry, biomarkers, etc.) are its ease of use, short measuring time, and non-invasiveness. Additionally, because it is small and mobile and quantifies continuous parameters, BIA is suitable for detecting changes over time at the outpatient clinic.

However, the one-cylinder BIA model could underestimate total body water (TBW) in ADPKD patients because they have cystic fluids in the abdomen [20]. To overcome this, we employed segmental BIA with a 5-cylinder model that considers the upper extremities (UE), lower extremities (LE), and trunk as 5 separate cylinders for independent impedance measurements. Using segmental BIA, we were able to obtain segmental data for ECW/TBW, lean mass (LM), and PhA from each of the 4-extremities and the trunk separately.

In our previous study, ADPKD patients, especially those with large abdominal cystic organ volume, are found to be at risk for malnutrition [10]. If the height-adjusted total kidney and liver volume (htTKLV), the sum of height-adjusted TKV (htTKV) and height-adjusted TLV (htTLV), is ≥2,340 (mL/m), the risk of malnutrition increases 8.7 times after adjusting for renal function [10]. This is a follow-up study to evaluate segmental BIA as a nutritional assessment tool for ADPKD patients. First, we assessed the relationships of various BIA parameters with SGA to determine the most suitable BIA parameters to reflect nutritional status in ADPKD patients. Second, we compared the BIA data of ADPKD patients with those of healthy subjects from the general population. Thirdly, to analyze the effect of abdominal cystic mass on malnutrition in ADPKD patients, we obtained the htTKV and htTLV of all patients using computed tomography (CT) and analyzed the effect on segmental BIA data. We performed this study among pre-dialysis patients with stage 1 to 4 CKD to exclude the effect of severe renal dysfunction on nutritional status.

## Materials and methods

This is a cross-sectional study of ambulatory patients who visited the outpatient ADPKD clinic following HOPE-PKD (coHOrt for genotype-PhenotypE correlation in ADPKD) protocol of Seoul National University Hospital. In the HOPE-PKD, a total of Korean 288 ADPKD patients were registered during December 2013 to March. 2014. Inclusion criteria of HOPE-PKD was subjects of 18 years or older and satisfied the Unified Criteria. The nutritional status of ADPKD patients were evaluated using SGA and BIA in our outpatient PKD clinic per standardized protocols. Patients with active cancer, infection, or renal replacement therapy or with a history of liver resection or transplantation were excluded. Detailed clinical information and reasons for liver resection or transplantation are discussed in a previous publication [21].

A database of BIA profiles from healthy subjects in the general population (Inbody Co., Ltd.) was used to compare with ADPKD patients after 1:1 matching. Data for 281 healthy subjects who were matched with the case patients for sex, age, and height ±2 cm were extracted from the database of the healthy population. PhA data was not included for the healthy population.

We used modified SGA with a seven-point scale, which has been validated in various studies in CKD patients [22]. Detailed nutritional assessment using modified SGA was described in our previous report [10]. The seven-point scale reflecting the dietician’s subjective judgment of the patient’s overall nutritional status was interpreted as follows: 7, well-nourished; 6, at risk; 5, mildly malnourished; 3–4, moderately malnourished; and 1–2, extremely malnourished.

Inbody S10 (Inbody Co., Ltd, Seoul, Korea), a multi-frequency segmental BIA analysis instrument, was used in this study [23]. In the outpatient’s clinic, the measurements were performed in the standing position with 4-electrodes connected to both hands and feet of patient’s. The BIA data of body fluid status, body composition parameters, and PhA of 50kHz were collected. In this study, we adjusted several quantitative segmental BIA parameters of the TBW, intracellular water (ICW), extracellular water (ECW), fat free mass (FFM), LM and fat mass (FM) by height, respectively (TBW/Ht, ICW/Ht, ECW/Ht, FFM/Ht, LM/Ht and FM/Ht). UE segmental parameters were defined as the average of the left and right arm data, and LE segmental parameters were defined in the same manner. Whole-body PhA (PhA_WB_) was defined as the PhA on the right-side of the body and was calculated from right-side body impedance, as described previously [24].

In our ADPKD clinic, abdominal CT scans were performed every other year [25], and the most recent abdominal CT scan was used to measure htTLV and htTKV. Total liver volume was calculated as the sum of the products of slice thickness and area measured on a set of contiguous image using Rapidia 2.8 CT software (INFINITT Healthcare Co. Ltd, Seoul, Korea) and the total kidney volume was estimated with the ellipsoid method [26]. The htTKLV, was defined as the sum of htTLV and htTKV. [10].

Anthropometric measurements (height, weight, and body mass index (BMI)) and laboratory measurements (serum hemoglobin, creatinine, total protein, albumin, and total cholesterol) were performed on the same day as the SGA and BIA evaluation. The estimated glomerular filtration rate (eGFR) was calculated with the CKD Epidemiology Collaboration (CKD-EPI) equation [27]. Continuous variables including age, height, weight, body mass index, serum hemoglobin, eGFR, and albumin showed normal distributions and were presented as mean value and standard deviation. However, htTKV, htTLV and htTKLV showed a non-normal distribution, and were therefore expressed as median value and interquartile range (IQR).

Since no subject had an SGA score less than 4 in the outpatient environment, all patients were classified into three groups: mildly to moderately malnourished (SGA score of ≤5), at risk (SGA score of 6), and well-nourished (SGA score of 7). For statistical analysis, we used the linear association test or the Jonckheere-Terpstra test to evaluate the *P* for trend among the three SGA groups. The value of *P for trend* <0.05 were interpreted as statistically significant. In the comparison study with healthy population data, paired t-test was used for normally distributed variables and Wilcoxon signed rank test for non-normally distributed variables. Receiver operating characteristic (ROC) curve analysis was used to evaluate BIA parameters for their ability to discriminate malnourished patients from well-nourished patients and to discriminate patients with significant htTKLV, which is ≥ 2,400mL/m. The Youden index was used to determine the optimal cut-off value in MedCalc for Windows version 14 (MedCalc Software, Ostend, Belgium). Correlation analysis was performed to determine the relationships of BIA parameters with htTKLV. Due to its skewed distribution, htTKLV was natural log-transformed to ln htTKLV. Log odds graphs of definite malnutrition over normal nutrition with respect to various BIA parameters were plotted with STATA statistical software package version 13 (Stata Corp., College Station, TX, USA). All other statistical analyses were conducted with SPSS version 22 (IBM Corporation, Armonk, NY, USA).

To exclude the renal function effect on BIA result, subgroup analysis of early stage CKD patients (CKD stage 1-3a) was conducted. In this subgroup analysis, the BIA data was compared among SGA groups and the association between BIA parameters and abdominal cystic organ volume was also analyzed. Additionally, the adjusted log odds graph of definite malnutrition over normal nutrition with respect to various BIA parameters were plotted in whole study population after adjusted with age, hemoglobin level and renal function.

This study was approved by the Institutional Review Board of Seoul National University Hospital (IRB No; 1205-112-411 and 1407-083-594). All participants provided written informed consent.

## Results

### Baseline characteristics according to SGA score

In total, 288 patients were included in the analysis, of whom 138 (47.9%) were female (Table 1). The mean age of the total population was 48.3±12.2 years, and the mean BMI was 23.4±2.8 kg/m^2^. In terms of CKD stage, 52 (18.1%), 116 (40.3%), 53 (18.4%), 46 (16.0%), and 21 patients (7.3%) were in stages 1, 2, 3A, 3B, and 4, respectively. The mean eGFR value was 65.3±25.3 mL/min/1.73 m^2^. Two patients (0.7%) were moderately malnourished (SGA score of 4), 19 (6.6%) were mildly malnourished (SGA score of 5), 63 (21.9%) were at risk for malnutrition (SGA score of 6), and 204 (70.8%) were well-nourished (SGA score of 7). The distribution of SGA scores and the mean SGA score did not differ between the two genders. Older age, lower weight, and lower BMI were associated with lower SGA score (*P* for trend <0.001, 0.002, and 0.044, respectively). The distribution of CKD stages differed across the SGA score groups, and the mean eGFR was lower in subjects with lower SGA score (*P* for trend <0.001 for both).

**Table 1:** Baseline patient characteristics according to nutritional status as evaluated by SGA.

Parameters	SGA 4 and 5	SGA 6	SGA 7	Total	*P* for trend
Number of patients	21 (7.3%)	63 (21.9%)	204 (70.8%)	288	
Female	9 (42.9%)	37 (58.7%)	92 (45.1%)	138 (47.9%)	0.148
Age (years)	53.4 ± 11.1	52.7 ± 12.6	46.4 ± 11.7	48.3 ± 12.2	**<0.001**
Height (cm)	164.0 ± 7.9	163.4 ± 7.9	167.6 ± 9.8	166.4 ± 9.5	**0.002**
Weight (kg)	59.1 ± 8.7	62.3 ± 9.7	67.0 ± 12.3	65.4 ± 11.8	**0.001**
BMI (kg/m^2^)	22.0 ± 2.7	23.3 ± 2.6	23.7 ± 2.9	23.4 ± 2.8	**0.044**
Hemoglobin (g/dL)	12.8 ± 1.1	13.2 ± 1.4	13.7 ± 1.5	13.5 ± 1.5	**<0.001**
Serum creatinine (mg/dL)	1.5 ± 0.6	1.4 ± 0.6	1.2 ± 0.6	1.2 ± 0.6	**0.001**
eGFR (mL/min/1.73 m^2^)	51.3 ± 23.2	57.1 ± 24.9	69.2 ± 24.6	65.3 ± 25.3	**<0.001**
Albumin (g/dL)	4.4 ± 0.3	4.3 ± 0.3	4.4 ± 0.4	4.4 ± 0.3	0.377
htTKLV (mL/m)	2,622 [1,719–4,906]	2,147 [1,535–2,687]	1,649 [1,311–2,151]	1,776 [1,361–2,381]	**<0.001**
htTKV (mL/m)	1, 282 [524–1,574]	903 [559–1,246]	640 [394–955]	697 [415–1,133]	**<0.001**
htTLV (mL/m)	1,298 [830–2,831]	1,023 [816–1,322]	957[819–1,147]	977 [819–1,196]	**0.036**

htTKLV, htTKV and htTLV are represented as median [interquartile value]. All other parameters are presented as mean ± standard deviation. BMI; body mass index, CKD; chronic kidney disease, eGFR; estimated glomerular filtration rates, htTKLV; height-adjusted total kidney and liver volume, htTKV; height-adjusted total kidney volume, htTLV; height-adjusted total liver volume, SGA, subjective global assessment

The median values of htTKLV, htTKV, and htTLV were 1,776 mL/m (IQR 1,361–2,381 mL/m), 697 mL/m (IQR 415–1,133 mL/m), and 977 mL/m (IQR 819–1,196 mL/m), respectively. Lower SGA scores corresponded to larger values of htTKLV (*P* for trend <0.001), htTKV (*P* for trend <0.001) and htTLV (*P* for trend = 0.036) (**Table 1**).

### Association of BIA parameters with SGA in ADPKD patients

**Fig 1:**
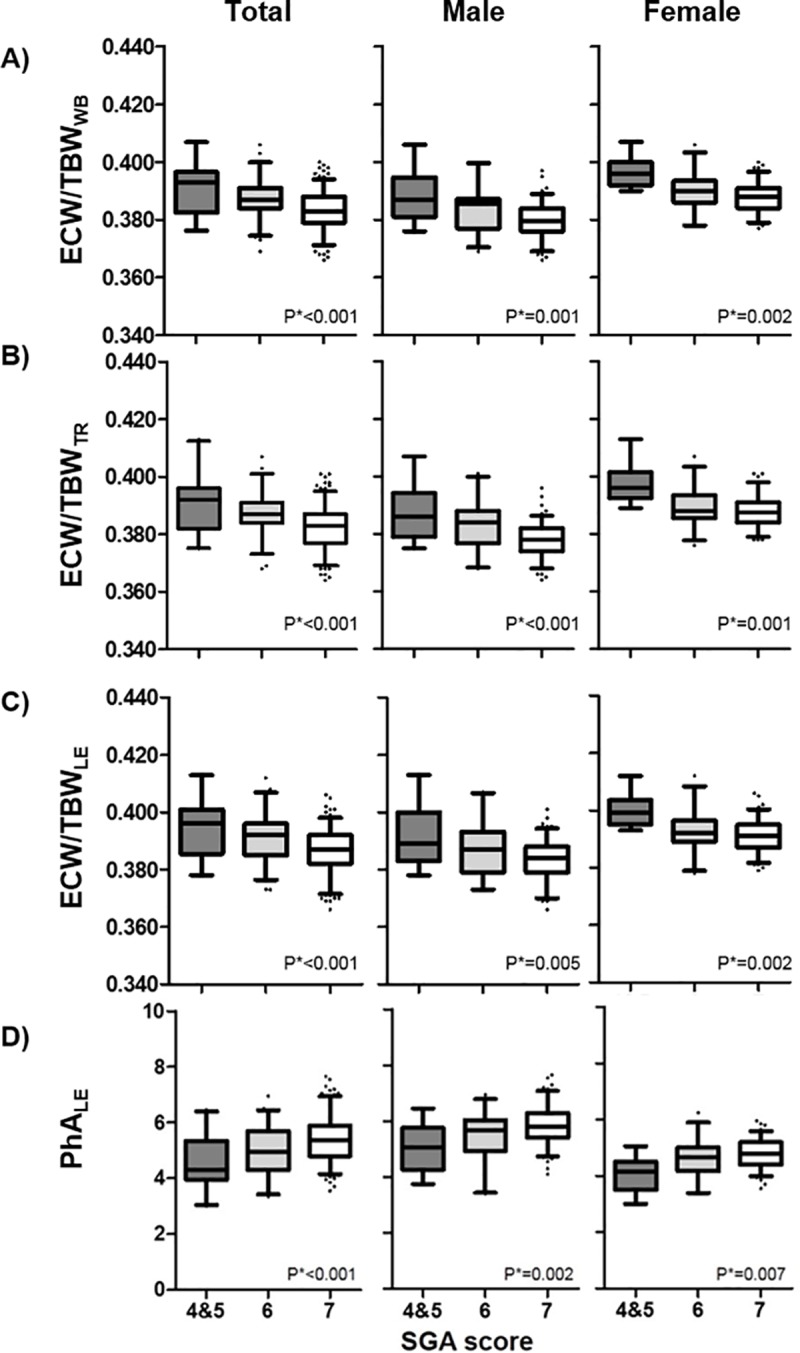
Associations of ECW/TBW and phase angle with SGA score. (A) ECW/TBW_WB_, (B) ECW/TBW_TR_, (C) ECW/TBW_LE_ and (D) PhA_LE._ECW/TBW_WB, TR_ and _LE_ and PhA_LE_ associated with SGA scores in both genders. *P; *P* for trends, SGA 7, well-nourished; SGA 6, at risk; SGA 5, mildly malnourished; SGA 3–4, moderately malnourished, ECW/TBW_WB_; ratio of extracellular water to total body water of whole-body, ECW/TBW_TR_; ratio of extracellular water to total body water of trunk, ECW/TBW_LE_; ratio of extracellular water to total body water of lower extremities, PhA_LE_; phase angle of lower extremities, SGA; subjective global assessment.

When we analyzed segmental BIA parameters across SGA score groups (4 or 5 vs. 6 vs. 7), the groups with lower SGA scores exhibited higher ECW/TBW_WB_, ECW/TBW_TR_, and ECW/TBW_LE_ (all for *P* for trend <0.001). The *P* for trend was statistically significant for UE ECW/TBW (ECW/TBW_UE_), but the trend was less robust than in the other segments of the body (*P* for trend = 0.035) (**Table 2, Fig 1)**.

**Table 2:** Association of segmental BIA parameters with SGA scores.

Parameters	Male				Female				Total				
	SGA ≤5	SGA 6	SGA 7	P for trend	SGA ≤5	SGA 6	SGA 7	P for trend	SGA ≤5	SGA 6	SGA 7	Total	*P* for trend
Number of patients	12	26	112		9	37	92		21	63	204	288	
ECW/TBW_WB_	0.388 ± 0.009	0.384 ± 0.008	0.380 ± 0.006	**0.001**	0.396 ± 0.005	0.390 ± 0.006	0.388 ± 0.005	**0.002**	0.391 ± 0.009	0.387 ± 0.007	0.383 ± 0.007	0.384 ± 0.007	**<0.001**
ECW/TBW_TR_	0.387 ± 0.010	0.383 ± 0.009	0.378 ± 0.006	**<0.001**	0.398 ± 0.007	0.390 ± 0.007	0.388 ± 0.005	**0.001**	0.391 ± 0.01	0.387 ± 0.008	0.382 ± 0.007	0.384 ± 0.008	**<0.001**
ECW/TBW_UE_	0.378 ± 0.004	0.378 ± 0.005	0.377 ± 0.004	0.051	0.381 ± 0.002	0.379 ± 0.003	0.379 ± 0.003	0.664	0.379 ± 0.004	0.379 ± 0.004	0.378 ± 0.004	0.378 ± 0.004	**0.035**
ECW/TBW_LE_	0.391 ± 0.010	0.388 ± 0.010	0.383 ± 0.007	**0.005**	0.400 ± 0.006	0.393 ± 0.007	0.391 ± 0.006	**0.002**	0.395 ± 0.010	0.391 ± 0.008	0.387 ± 0.007	0.388 ± 0.008	**<0.001**
PhA_WB_ (θ)	5.3 ± 0.7	5.3 ± 0.9	5.4 ± 0.7	0.530	5.5 ± 0.9	5.3 ± 0.8	5.4 ± 0.7	0.753	5.4 ± 0.8	5.3 ± 0.8	5.4 ± 0.7	5.4 ± 0.7	0.478
PhA_TR_ (θ)	8.8 ± 1.4	9.3 ± 1.5	9.5 ± 1.2	0.053	8.4 ± 1.4	8.3 ± 1.0	8.4 ± 1.2	0.480	8.6 ± 1.4	8.6 ± 1.3	9.0 ± 1.3	8.9 ± 1.3	**0.015**
PhA_UE_ (θ)	5.2 ± 0.5	5.5 ± 0.6	5.7 ± 0.5	**<0.001**	4.6 ± 0.2	4.7 ± 0.4	4.7 ± 0.4	0.150	4.9 ± 0.5	5.0 ± 0.6	5.3 ± 0.7	5.2 ± 0.7	**<0.001**
PhA_LE_ (θ)	5.0 ± 0.9	5.4 ± 0.9	5.8 ± 0.7	**0.002**	4.0 ± 0.6	4.6 ± 0.6	4.8 ± 0.5	**0.007**	4.6 ± 0.9	5.0 ± 0.8	5.4 ± 0.8	5.2 ± 0.9	**<0.001**

All parameters are presented as mean ± standard deviation. BIA; bioelectrical impedance analysis, ECW/TBW_WB_; ratio of extracellular water to total body water of whole-body, ECW/TBW_TR_; ratio of extracellular water to total body water of trunk, ECW/TBW_UE_; ratio of extracellular water to total body water of upper extremities, ECW/TBW_LE_; ratio of extracellular water to total body water of lower extremities, PhA_WB_; Phase angle of whole-body, PhA_TR_; Phase angle of trunk, PhA_UE_; Phase angle of upper extremities, PhA_LE_; Phase angle of lower extremities, SGA; subjective global assessment

Segmental PhA values also correlated with SGA score. The UE PhA (PhA_UE_, *P* for trend <0.001) and PhA_LE_ (*P* for trend <0.001) differed significantly among the three SGA groups, with the mean value decreasing as the SGA score decreased. The *P* for trend was also significant for the trunk PhA (PhA_TR_) across the SGA score groups, but the PhA_TR_ trend was less robust than in the other segments of the body (*P* for trend = 0.015). The PhA_WB_, which has been reported as a nutritional marker in previous studies in other disease conditions [24], did not differ significantly among the three SGA groups (*P* for trend = 0.478) (**Table 2**).

For each body fluid parameter, such as ICW/Ht, ECW/Ht, TBW/Ht, the *P* for trend was statistically significant, but the mean values failed to exhibit definite trends across the SGA score groups. Regarding body composition parameters, the FFM/Ht and the LM/Ht for the WB, UE, and LE exhibited statistically significant *P* for trend values (all *P* for trends <0.01) without definite trends in the mean values across the SGA groups. FM/Ht did not differ significantly among the three groups. Only the trunk LM/Ht (LM/Ht_TR_) exhibited a definite trend of decreasing mean value with decreasing SGA score (*P* for trend = 0.002) (**S1 Table and S1 Fig and S2 Fig**).

### Differences between the genders in the association of BIA parameters with SGA

In our previous study [10], several parameters that were associated with SGA score in males (such as age, body weight, BMI, and hemoglobin level) showed no association in females. Therefore, in this study, subgroup analyses were undertaken to determine whether there were gender differences in the associations of BIA parameters with SGA score.

Four parameters of higher ECW/TBW_WB_, ECW/TBW_TR_, and ECW/TBW_LE_ and lower PhA_LE_ values correlated with lower SGA score in both male and female ADPKD. However, the other body fluid parameters and body composition parameters correlated with SGA score only in the male subgroup. ECW/TBW_UE_ and PhA_TR_ values, for which the *P* for trend values were statistically significant in the total population, did not exhibit such trends in either gender subgroup (**Table 2, S1 Table**).

### ROC curve analysis of BIA parameters with SGA in ADPKD patients

ROC curve analysis was conducted to identify the most suitable BIA parameter for predicting definite malnutrition (SGA ≤5). The parameters that differed significantly (*P* for trend <0.05) in both male and female patients and in the total population were analyzed, and ROC curves were generated using the data from subjects with SGA score of 7 (well-nourished) or ≤5 (malnutrition).

**Fig 2:**
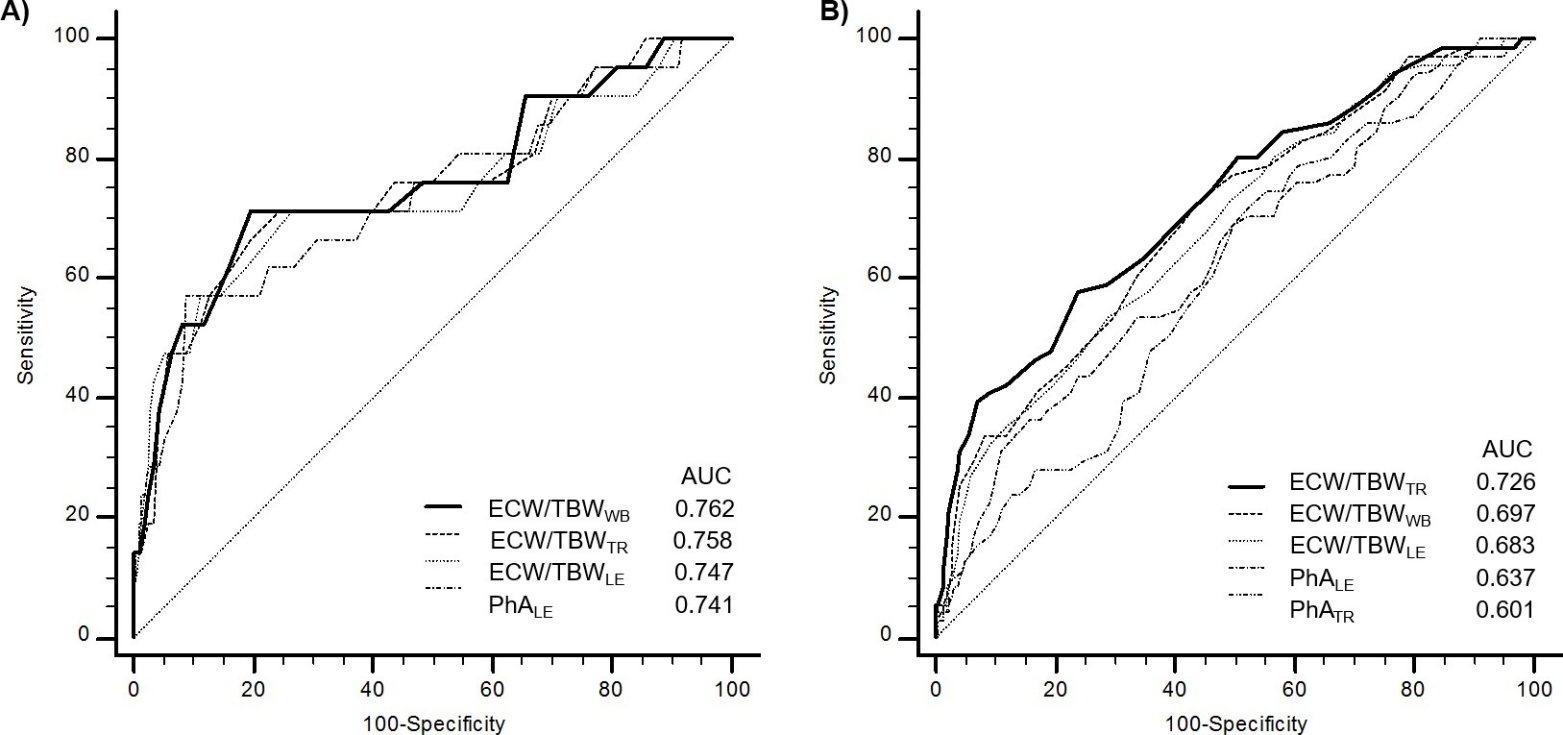
ROC curve of BIA parameters for the diagnosis of malnutrition and significant htTKLV. Predicting A) malnutrition defined as SGA score of 4 and 5 and B) significant htTKLV of ≥2,400 mL/m using BIA parameters. In the ROC curve analysis of BIA parameters to predict malnutrition, all AUCs were larger than 0.7 and the AUC for ECW/TBW_WB_ was the largest (0.762). In the ROC curve analysis of BIA parameters to predict significant htTKLV values of ≥2,400 mL/m, all AUCs of parameters shown in the figure were larger than 0.6 and the AUC for ECW/TBW_TR_ was the largest (0.726). AUC; area under curve, BIA; bioelectrical impedance analysis, ECW/TBW_WB_; ratio of extracellular water to total body water of whole-body, ECW/TBW_TR_; ratio of extracellular water to total body water of trunk, ECW/TBW_LE_; ratio of extracellular water to total body water of lower extremities, PhA_TR_; Phase angle of trunk, PhA_LE_; Phase angle of lower extremities, ROC; receiver-operating characteristics, SGA; subjective global assessment.

The areas under the curve (AUCs) and the ROC curves of the four relevant parameters are shown in **Fig 2(A)**. The AUC for ECW/TBW_WB_ was the largest (0.762), and the AUCs for all the other parameters were also greater than 0.7, in the following order: ECW/TBW_TR_ (0.758), ECW/TBW_LE_ (0.747) and PhA_LE_ (0.741).

### Log odds analysis of BIA parameters to predict malnutrition in ADPKD patients

**Fig 3:**
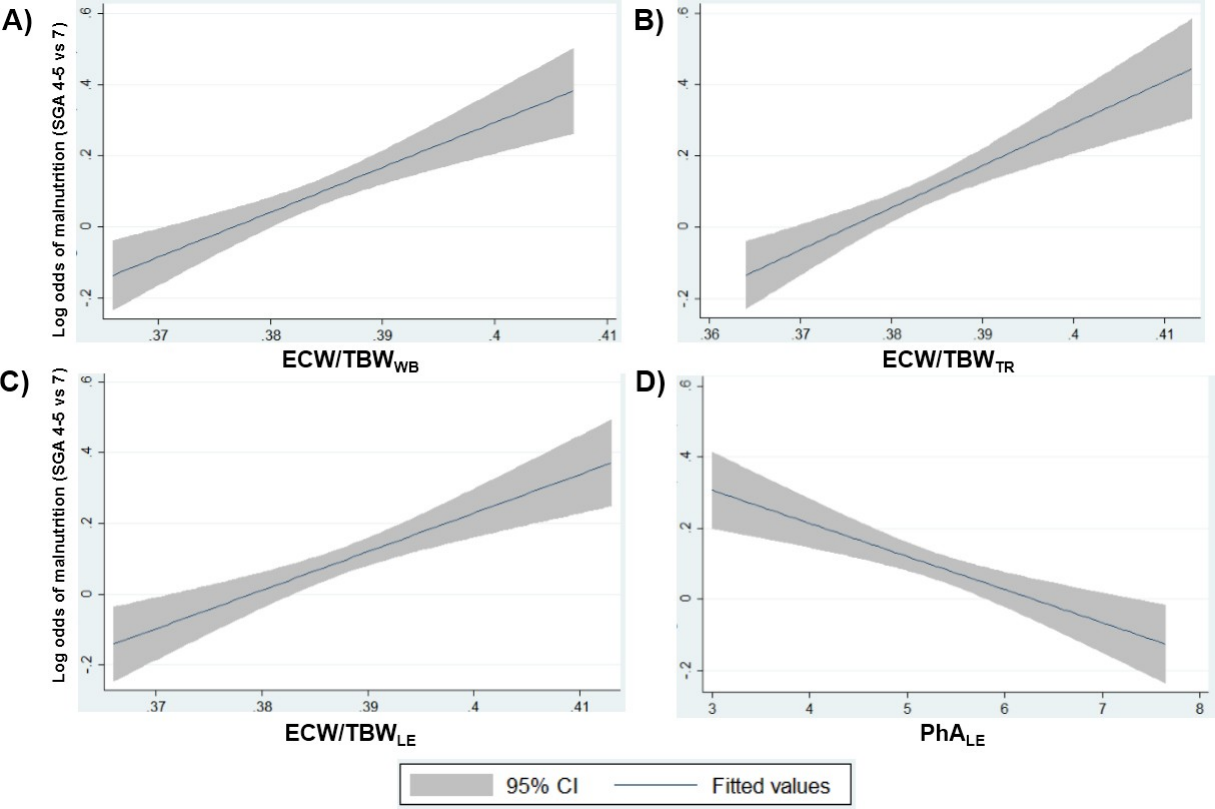
Likelihood (log odds) of having malnutrition (SGA 4&5 vs 7) with respect to various BIA parameters. A) ECW/TBW_WB_, (B) ECW/TBW_TR_, (C) ECW/TBW_LE_ and (D) PhA_LE_ (θ). Log odds of having malnutrition increased as the ECW/TBW _WB_, _TR_ and _LE_ increased. On the contrary, log odds decreased with PhA_LE_ increased. BIA; bioelectrical impedance analysis, ECW/TBW_WB_; ratio of extracellular water to total body water of whole-body, ECW/TBW_TR_; ratio of extracellular water to total body water of trunk, ECW/TBW_LE_; ratio of extracellular water to total body water of lower extremities, PhA_LE_; Phase angle of lower extremities, SGA; subjective global assessment.

When the log odds of malnutrition (SGA score of ≤5) were plotted across the values of each of the four BIA parameters that were significant in each sex and in the total population, the mean (95% confidence interval) log odds increased significantly as the ECW/TBW_WB_, ECW/TBW_TR_, and ECW/TBW_LE_ increased. On the contrary, PhA_LE_ correlated negatively with log odds (**Fig 3**). The cut-off values for positive likelihood of malnutrition were ECW/TBW_WB_ >0.380, ECW/TBW_TR_ >0.379, ECW/TBW_LE_ >0.383 and PhA_LE_ <5.8. The same trends were observed in the subgroup analysis of both males and females (**Table 3**). Similar log odds graphs were obtained after adjustment for age, hemoglobin level, and renal function (**S3 Fig**).

**Table 3:** Cutoff value for positive log odds of having malnutrition with respect to various BIA parameters.

	Total	Male	Female
**ECW/TBW**_**WB**_	> 0.380	> 0.378	>0 .387
**ECW/TBW**_**TR**_	> 0.379	> 0.376	> 0.387
**ECW/TBW**_**LE**_	> 0.383	> 0.381	> 0.390
**PhA**_**LE**_ **(**θ)	< 5.8	< 6.1	< 4.9

BIA; bioelectrical impedance analysis, ECW/TBW_WB_; ratio of extracellular water to total body water of whole-body, ECW/TBW_TR_; ratio of extracellular water to total body water of trunk, ECW/TBW_LE_; ratio of extracellular water to total body water of lower extremities, PhA_LE_; Phase angle of lower extremities, SGA; subjective global assessment

### Differences in BIA parameters between the healthy population and ADPKD patients

To characterize the BIA parameters in ADPKD, we compared the BIA data of ADPKD patient with healthy population’s by extracting 1:1 matched, 281 healthy subjects from the database of Inbody Co., Ltd, [15]. After the subjects were matched for sex, age, and height ±2 cm, there was no significant difference in weight or BMI between the ADPKD and healthy groups (p = 0.086 and 0.088, respectively).

The mean values of body fluid parameters including ICW/Ht, ECW/Ht, and TBW/Ht were higher in ADPKD patients than in the general population (*P*<0.001 for all). The mean values of ECW/TBW_WB_, ECW/TBW_TR_, and ECW/TBW_LE_ were higher in ADPKD patients than in healthy subjects (*P*<0.001 for all).

Among the body composition parameters, FFM/Ht was higher in ADPKD patients than in healthy subjects and FM/Ht was significantly lower in ADPKD patients (*P*<0.001 for both). Whole-body LM/Ht (LM/Ht_WB_) and lower extremity LM/Ht (LM/Ht_LE_) were also higher in ADPKD patients than in the healthy population (*P*<0.001 for both). Increased LM/Ht_WB_ and LM/Ht_LE_ might reflect the edematous condition in ADPKD resulted from the decreased renal function and/or inferior vena cava compression by cystic organs. On the contrary, trunk LM/Ht (LM/Ht_TR_) was lower in ADPKD patients (*P*<0.001). (**Table 4**).

**Table 4:** Comparison of BIA parameter s between ADPKD patients and healthy population control.

	Total (n = 281)		
	ADPKD patients	Healthy population	*P*-value
Age (years)	48.4 ± 11.9	48.4 ± 11.9	0.385
Height (cm)	64.9 ± 11.3	65.2 ± 10.6	0.230
Weight (kg)	166.0 ± 9.0	165.9 ± 9.0	0.086
BMI (kg/m^2^)	23.4 ± 2.8	23.6 ± 2.5	0.088
ICW/Ht (L/m)	13.6 ± 2.2	13.2 ± 2.1	**<0.001**
ECW/Ht (L/m)	8.5 ± 1.2	8.2 ± 1.2	**<0.001**
TBW/Ht (L/m)	22.1 ± 3.4	21.4 ± 2.3	**<0.001**
ECW/TBW_WB_	0.385 ± 0.007	0.382 ± 0.007	**<0.001**
ECW/TBW_TR_	0.384 ± 0.008	0.382 ± 0.007	**<0.001**
ECW/TBW_UE_	0.378 ± 0.004	0.378 ± 0.005	0.085
ECW/TBW_LE_	0.388 ± 0.008	0.384 ± 0.008	**<0.001**
FM/Ht (kg/m)	8.8 ± 3.4	10.1 ± 3.2	**<0.001**
FFM/Ht (kg/m)	30.1 ± 4.6	29.1 ± 4.4	**<0.001**
LM/Ht_WB_ (kg/m)	28.3 ± 4.4	27.5 ± 4.2	**<0.001**
LM/Ht_TR_ (kg/m)	12.9 ± 2.0	13.2 ± 2.0	**<0.001**
LM/Ht_UE_ (kg/m)	3.1 ± 0.7	3.1 ± 0.6	0.447
LM/Ht_LE_ (kg/m)	9.4 ± 1.7	8.9 ± 1.5	**<0.001**

All parameters are presented as mean ± standard deviation. BIA; bioelectrical impedance analysis, ECW/TBW_WB_; ratio of extracellular water to total body water of whole-body, ECW/TBW_TR_; ratio of extracellular water to total body water of trunk, ECW/TBW_UE_; ratio of extracellular water to total body water of upper extremities, ECW/TBW_LE_; ratio of extracellular water to total body water of lower extremities, FM/Ht; height-adjusted fat mass, FFM/Ht; height-adjusted fat free mass, LM/Ht_WB_; height-adjusted lean mass of whole-body, LM/Ht_TR_; height-adjusted lean mass of trunk, LM/Ht_UE_; height-adjusted lean mass of upper extremities, LM/Ht_LE_; height-adjusted lean mass of lower extremities, SGA; subjective global assessment

### Subgroup analysis of early-stage CKD patients

We conducted subgroup analysis (n = 221 patients) of subjects with CKD stages 1 to 3a to exclude the effects of uremia on the nutritional and fluid status of ADPKD patients. The results of the subgroup analysis were similar to those of the overall analysis. (**S2 Table and S3 Table**)

### Associations between BIA parameters and abdominal cystic organ volume (htTKLV)

**Fig 4:**
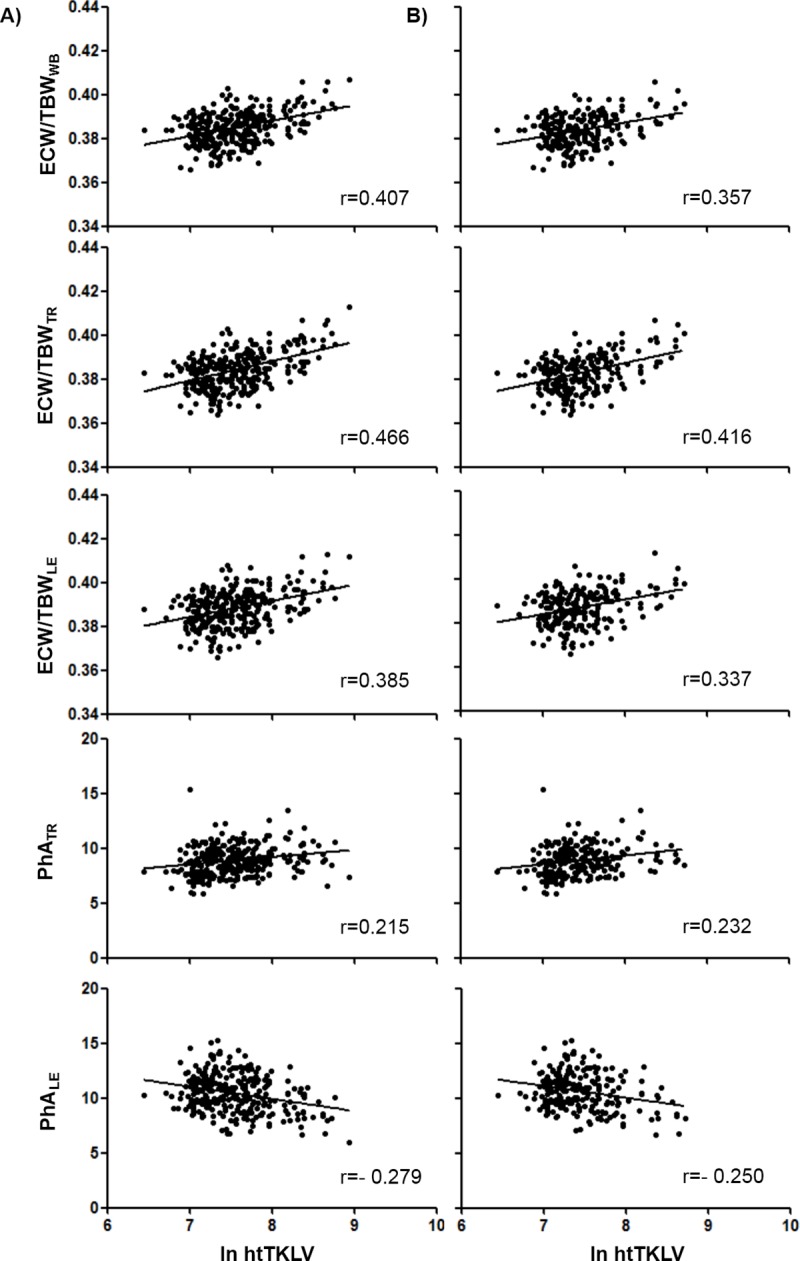
Scatter plot and regression line of BIA parameters with ln htTKLV. Scatter plot of BIA parameters with ln htTKLV in total population and (B) in eGFR ≥45 mL/min/1.73 m^2^ patients. ECW/TBW_WB_, _TR_ and,_LE_ and PhA_TR_ showed positive correlation with ln htTKLV. On the contrary, PhA_LE_ showed negative correlation with ln htTKLV. ECW/TBW TR showed highest correlation with ln htTKLV (r = 0.466) BIA; bioelectrical impedance analysis, ECW/TBW_WB_; ratio of extracellular water to total body water of whole-body, ECW/TBW_TR_; ratio of extracellular water to total body water of trunk, ECW/TBW_LE_; ratio of extracellular water to total body water of lower extremities, eGFR; estimated glomerular filtration rates, ln htTKLV; natural log value of height-adjusted total kidney and liver volume, PhA_TR_; Phase angle of trunk, PhA_LE_; Phase angle of lower extremities.

We used segmental BIA data to elucidate the correlations of various BIA parameters with htTKLV. **Fig 4** displays the scatter plots and Pearson’s correlation coefficients for the relationships between ln htTKLV and BIA parameters such as the ECW/TBW_TR_, ECW/TBW_WB_ and ECW/TBW_LE_, PhA_TR_ and PhA_LE_. The highest correlation was observed for the ECW/TBW_TR_ (r = 0.466), followed by ECW/TBW_WB_ (r = 0.407), ECW/TBW_LE_ (r = 0.385), and PhA_TR_ (r = 0.215). The PhA_LE_ correlated negatively with ln htTKLV (r = -0.279). In the subgroup analysis of patients with eGFR ≥45 mL/min/1.73 m^2^, the correlations of ln htTKLV with various BIA parameters in this subgroup were similar to those in the total population. The highest correlation was still noticed for ECW/TBW_TR_ (r = 0.416), followed by ECW/TBW_WB_ (r = 0.357), ECW/TBW_LE_ (r = 0.337), PhALE (r = -0.250) and PhA_TR_ (r = 0.232) in the subgroup analysis of patients with eGFR ≥45 mL/min/1.73 m^2^ (Fig 4B).

ROC curve analysis was conducted to identify the most suitable BIA parameter for predicting significant htTKLV values of ≥2,400 mL/m. From previous study, htTKLV values ≥2,340 mL/m increased malnutrition risk 8.7 time [10]. In this study, to simplify the number, we used the cut-off value for significant htTKLV as 2,400 mL/m. ECW/TBW_TR_ showed the largest AUC (0.726) with the following order; ECW/TBW_WB_ (0.697), ECW/TBW_LE_ (0.683), PhA_LE_ (0.637) and PhA_TR_ (0.601). The cut off value of ECW/TBW_TR_ to predict significant htTKLV values ≥ 2,400 mL/m was 0.387 with sensitivity of 57.7% and specificity of 76%. (**Fig 2(B))**

## Discussion

In a previous study, we found that even in the early stages of CKD, about 30% of patients had SGA score below 6, and that an increased htTKLV was an independent risk factor for malnutrition in ADPKD patients [10]. Therefore, nutritional assessment is important in ADPKD patients, especially for those with large abdominal volume [12]. In addition to SGA, BIA can be used as a nutritional assessment tool, with the advantage of having objective, continuous variables. In ADPKD, with cystic organs, conventional one-cylinder-model BIA might underestimate the TBW, due to the truncal geometry and tissue interfacing [20]. To overcome the limitation of one-cylinder-model BIA in the use of ADPKD patients, we evaluated segmental BIA, which is based on 5-cylinder model, as a quantitative tool for nutritional assessment in ADPKD patients in this study.

The ECW/TBW_WB_, ECW/TBW_TR_, and ECW/TBW_LE_ and PhA_LE_ correlated significantly with SGA score of ADPKD patients. Even though the difference was minimal, ECW/TBW_WB_ showed the best correlation with the SGA score. These findings correspond to the results of other studies in various clinical settings. ECW/TBW, which reflects an edematous status and/or malnutrition, is an important clinical factor related with the outcomes of CKD, liver cirrhosis, heart failure, and intensive care unit patients [28–31]. PhA, defined as the vector angle difference between the resistance (R) and reactance (Xc), reflects cell viability and membrane stability and is also known as a useful nutritional marker. Reduced PhA values are associated with malnutrition and poor outcomes in patients with CKD, cancer, and liver cirrhosis [32–35]. Our results indicated that high ECW/TBW and low PhA are clinically useful BIA parameters for the detection of malnutrition in ADPKD patients. (**Fig 3**).

Decreased PhA has been regarded as a one of the best nutritional marker. However, except PhA_LE_, none of other segmental PhA parameters showed any correlation with the nutritional status in both gender of ADPKD patients. These results could represent the impact of organomegaly, since PhA_TR_ was positively correlated with htTKLV. Therefore, in ADPKD patients, among segmental PhA parameters, only PhA_LE,_ where the effect of organomegaly are minimal are suitable for nutritional parameters.

Since segmental BIA can measure the segmental fluid volume, higher ECW/TBW_TR_ was positively associated with htTKLV. Other parameters such as ECW/TBW_WB,_ ECW/TBW_LE,_ and PhA_TR_ also showed positive correlations and PhA_LE_ demonstrated negative correlations with htTKLV (**Fig 4**). Our previous study showed that htTKLV was an independent risk factor for malnutrition in ADPKD [10]. ROC analysis showed that ECW/TBW_TR_ of > 0.387 was related to significant htTKLV ≥2,400 mL/m. These data suggested that ECW/TBW_TR_ could reflect nutritional status in ADPKD patients mainly caused by the mass effect of organomegaly.

Deterioration of renal function is another significant factor of malnutrition in ADPKD. To exclude effect of reduced renal function on nutritional status and BIA parameters, we created an adjusted likelihood graph. After adjustment for age, hemoglobin level, and CKD stage, higher ECW/TBW_WB_, ECW/TBW_TR_, and ECW/TBW_LE_ and lower PhA_LE_ values were associated with a greater likelihood of malnutrition (**S3 Fig**). Also in the subgroup analysis with CKD in stages 1-3a population, the correlations of BIA parameters with SGA scores were similar to those in the total population (**S2 Table and S3 Table**). This implies that, aside from reduced renal function, an increased htTKLV influences the nutritional status of ADPKD patients independently, and that segmental BIA can detect this malnutrition even in early-stage CKD patients. BIA parameters were also directly associated with ln htTKLV in ADPKD patients even in early CKD stages (**Fig 2(B)**). An increased htTKLV might affect BIA parameters by causing pressure-related gastrointestinal symptoms that lead to malnutrition [9]. Other pressure-related complications such as caval compression and LE edema could also influence BIA results.

The body composition parameters did not exhibit trends of association with SGA score in ADPKD population as in other CKD patients [36]. In the male subgroup, all the body composition parameters had significant positive correlations with the SGA score, while none were meaningful in females. This discrepancy between the genders could be related to the lower muscle mass in females than in males. Since our study was undertaken in Koreans, and in an outpatient setting where only mild to moderate malnourishment was observed, the degree of sarcopenia might have been too mild to be detected with statistical significance. Also ECW increase in CKD patients due to sodium retention and volume overload can result in overestimation of LM measured by BIA adopting 2-compartment body composition model (FFM and FM) [37, 38]. However in the analysis of the study patients to see the association between ECW/TBW and skeletal muscle index, defined as the sum of appendicular lean mass divided by height square, the majority of patients were in the range of normal volume status (ECW/TBW between 0.36–0.40) and non-sarcopenic status suggesting the minimal effect of volume overload on LM value from BIA in our study patients [39, 40]. (**S4 Fig**)

It was previously unknown how polycystic masses would be presented in BIA, therefore we compared the BIA results between ADPKD patients and the healthy population. Greater values for body fluid parameters and ECW/TBW_WB_, ECW/TBW_TR_, and ECW/TBW_LE_ were observed in the ADPKD group than in the healthy population. Increased ECW/TBW_WB_ and ECW/TBW_TR_ in ADPKD may demonstrated the influence of fluid-filled cysts in the abdominal organs. In case of ECW/TBW_LE_, where the effect of abdominal cystic mass were minimal, may represent nutritional status per se. However, compression effect of organomegaly on intra-abdominal vessels which cause LE edema in ADPKD also participate in the increment of ECW/TBW_LE_.

This study has a few limitations. Since this study was conducted in an outpatient clinic, patients with severe malnutrition have been excluded. Also there was no control subjects with CKD other than ADPKD. The Kidney and liver volumes were measured using CT instead of MRI. Additionally, information on the menstrual cycle in females was not gathered. Even though, this is the first study to evaluate BIA as a nutritional assessment tool in ADPKD patients and to identify the correlation of BIA parameters with abdominal cystic organ volume. In addition, the strength of the present study is that the kidney and liver volumes were measured in every patient to characterize their effect on the nutritional status and their relationship with segmental BIA parameters in ADPKD patients. Follow up studies to determine the association of malnutrition with clinical outcomes are needed. Using other malnutrition criteria such as malnutrition inflammation score [13] and newly developed GLIM criteria [41] would provide more information on the nutritional status in ADPKD patients. Studies of other nutritional biomarkers and body composition measures using a prospective cohort would enhance our understanding and management of malnutrition in ADPKD patients.

In conclusion, the ECW/TBW_WB_ and other BIA parameters of ECW/TBW_TR_, ECW/TBW_LE_ and PhA_LE_ were qualified malnutrition parameters. The ECW/TBW_TR_ was not only associated with malnutrition, but also with htTKLV. Other parameters such as ECW/TBW_WB_, ECW/TBW_LE_ and PhA_LE_ also showed correlation with htTKLV but with less significance.

## Conclusions

These results showed that segmental BIA can be a suitable tool for assessing nutritional status as well as the impact of abdominal cystic organs in ADPKD patients, which provides continuous and segmental parameters.

## Supporting information

S1 Table Association of BIA parameters with SGA scores according to gender. (DOCX)

S2 Table Baseline patient characteristics according to nutritional status as evaluated by SGA in subgroup analysis among CKD stage 1-3A. (DOCX)

S3 Table Association of BIA parameters with SGA scores among CKD stage 1-3A. (DOCX)

S1 Fig Association of Body fluid parameters with SGA scores. (A) ICW/Ht (L/m), (B) ECW/Ht (L/m), (C) TBW/Ht (L/m).
ICW/Ht, ECW/Ht and TBW/Ht did not show significant association with SGA scores in female population.
*P; P for trends
SGA 7, well-nourished; SGA 6, at risk; SGA 5, mildly malnourished; SGA 3–4, moderately
ICW/Ht; height-adjusted intracellular water, ECW/Ht; height-adjusted extracellular water, TBW/Ht; height-adjusted total body water, SGA; subjective global assessment.
(TIF)

S2 Fig Association of body composition parameters with SGA scores. (A) FM/Ht_WB_ (kg/m), (B) LM/Ht_WB_ (kg/m), (C) LM/Ht_TR_ (kg/m), (D) LM/Ht_UE_ (kg/m), and (E) LM/Ht_LE_ (kg/m).
All body composition parameter did not show significant association with SGA scores in female population.
*P; *P* for trends
SGA 7, well-nourished; SGA 6, at risk; SGA 5, mildly malnourished; SGA 3–4, moderately
FM/Ht; height-adjusted fat mass, FFM/Ht; height-adjusted fat free mass, LM/Ht_WB_; height-adjusted lean mass of whole-body, LM/Ht_TR_; height-adjusted lean mass of trunk, LM/Ht_UE_; height-adjusted lean mass of upper extremities, LM/Ht_LE_; height-adjusted lean mass of lower extremities, SGA; subjective global assessment.
(TIF)

S3 Fig Adjusted Likelihood (log odds) of having malnutrition (SGA 4&5 vs 7) according to BIA parameters. A) ECW/TBW_WB_, (B) ECW/TBW_TR_, (C) ECW/TBW_LE_ and (D) PhA_LE_ (θ), after adjusted with age, hemoglobin and renal function
In the adjusted likelihood of having malnutrition showed positive correlation with ECW/TBW_WB_, _TR_ and _LE_ and negative correlation with PhA_LE_
BIA; bioelectrical impedance analysis, ECW/TBW_WB_; ratio of extracellular water to total body water of whole-body, ECW/TBW_TR_; ratio of extracellular water to total body water of trunk, ECW/TBW_LE_; ratio of extracellular water to total body water of lower extremities, PhA_LE_; Phase angle of lower extremities, SGA; subjective global assessment.
(TIF)

S4 Fig Association of ECW/TBW _WB_ with skeletal muscle index. The cutoff value of ECW/TBW for edema was 0.4 and skeletal muscle index for sarcopenia was a) 7.0 kg/m^2^ for male and 5.7 kg/m^2^ for female, respectively.
In the subject group with both ECW/TBW_WB_ > 0.4 and skeletal muscle index higher than the cutoff for sarcopenia, the lean mass could be overestimated because over-hydration are included in the lean mass. However, in our study population, majority of subjects were not included in this group suggesting the minimal effect of volume overload on lean mass measured by BIA.
BIA; bioelectrical impedance analysis, ECW/TBW _WB_; ratio of extracellular water to total body water of whole-body.
(TIF)

## References

[1] Pupim LB, Caglar K, Hakim RM, Shyr Y, Ikizler TA. Uremic malnutrition is a predictor of death independent of inflammatory status. Kidney Int. 2004;66(5):2054–60. 10.1111/j.1523-1755.2004.00978.x 15496179

[2] de Mutsert R, Grootendorst DC, Axelsson J, Boeschoten EW, Krediet RT, Dekker FW. Excess mortality due to interaction between protein-energy wasting, inflammation and cardiovascular disease in chronic dialysis patients. Nephrol Dial Transplant. 2008;23(9):2957–64. 10.1093/ndt/gfn167 18400817

[3] Carrero JJ, Stenvinkel P, Cuppari L, Ikizler TA, Kalantar-Zadeh K, Kaysen G, et al Etiology of the proteinenergy wasting syndrome in chronic kidney disease: a consensus statement from the International Society of Renal Nutrition and Metabolism (ISRNM). J Ren Nutr 2013; 23: 77–90. 10.1053/j.jrn.2013.01.001 23428357

[4] Lowrie EG, Huang WH, Lew NL. Death risk predictors among peritoneal dialysis and hemodialysis patients: a preliminary comparison. American journal of kidney diseases: the official journal of the National Kidney Foundation. 1995;26(1):220–8. 10.1016/0272-6386(95)90177-9 7611256

[5] Leavey SF, Strawderman RL, Jones CA, Port FK, Held PJ. Simple nutritional indicators as independent predictors of mortality in hemodialysis patients. American journal of kidney diseases: the official journal of the National Kidney Foundation. 1998;31(6):997–1006. 10.1053/ajkd.1998.v31.pm9631845 9631845

[6] Streja E, Molnar MZ, Kovesdy CP, Bunnapradist S, Jing J, Nissenson AR, et al Associations of pretransplant weight and muscle mass with mortality in renal transplant recipients. Clin J Am Soc Nephrol. 2011;6(6):1463–73. 10.2215/CJN.09131010 21415312 PMC3109945

[7] Molnar MZ, Kovesdy CP, Bunnapradist S, Streja E, Mehrotra R, Krishnan M, et al Associations of pretransplant serum albumin with post-transplant outcomes in kidney transplant recipients. American journal of transplantation: official journal of the American Society of Transplantation and the American Society of Transplant Surgeons. 2011;11(5):1006–15. 10.1111/j.1600-6143.2011.03480.x PMC3083471 21449945

[8] Clinical practice guidelines for nutrition in chronic renal failure. K/DOQI, National Kidney Foundation. Am J Kidney Dis. 2000;35(6 Suppl 2):S1–140. 10895784 10.1053/ajkd.2000.v35.aajkd03517

[9] Kim H, Park HC, Ryu H, Kim K, Kim HS, Oh KH et al Clinical Correlates of Mass Effect in Autosomal Dominant Polycystic Kidney Disease. PloS One. 2015;10(12):e0144526 10.1371/journal.pone.0144526 26641645 PMC4671651

[10] Ryu H, Kim H, Park HC, Kim H, Cho EJ, Lee KB et al Total kidney and liver volume is a major risk factor for malnutrition in ambulatory patients with autosomal dominant polycystic kidney disease. BMC Nephrol. 2017;18(1):22 10.1186/s12882-016-0434-0 28088190 PMC5237538

[11] Temmerman F, Missiaen L, Bammens B, Laleman W, Cassiman D, Verslype C, et al Systematic review: the pathophysiology and management of polycystic liver disease. Aliment Pharmacol Ther. 2011;34(7):702–13. 10.1111/j.1365-2036.2011.04783.x 21790682

[12] Cnossen WR, Drenth JP. Polycystic liver disease: an overview of pathogenesis, clinical manifestations and management. Orphanet J Rare Dis. 2014;9(1):69. 24886261 10.1186/1750-1172-9-69 PMC4030533

[13] Kalantar-Zadeh K, Kopple JD, Block G, Humphreys MH. A malnutrition-inflammation score is correlated with morbidity and mortality in maintenance hemodialysis patients. Am J Kidney Dis. 2001;38(6):1251–63. 10.1053/ajkd.2001.29222 11728958

[14] Lawson JA, Lazarus R, Kelly JJ. Prevalence and prognostic significance of malnutrition in chronic renal insufficiency. J Ren Nutr. 2001;11(1):16–22. 11172449 10.1016/s1051-2276(01)85914-8

[15] Barbosa-Silva MCG, Barros AJ. Bioelectrical impedance analysis in clinical practice: a new perspective on its use beyond body composition equations. Curr Opin Clin Nutr Metabo Care. 2005;8(3):311–7. 10.1097/01.mco.0000165011.69943.39 15809535

[16] Kyle UG, Genton L, Pichard C. Low phase angle determined by bioelectrical impedance analysis is associated with malnutrition and nutritional risk at hospital admission. Clin Nutr. 2013;32(2):294–9. 10.1016/j.clnu.2012.08.001 22921419

[17] Pirlich M, Schütz T, Spachos T, Ertl S, Weiss ML, Lochs H, et al Bioelectrical impedance analysis is a useful bedside technique to assess malnutrition in cirrhotic patients with and without ascites. Hepatology. 2000;32(6):1208–15. 10.1053/jhep.2000.20524 11093726

[18] Gupta D, Lis CG, Dahlk SL, King J, Vashi PG, Grutsch JF, et al The relationship between bioelectrical impedance phase angle and subjective global assessment in advanced colorectal cancer. Nutr J. 2008;7(1):19. 18590554 10.1186/1475-2891-7-19 PMC2483715

[19] Tai R, Ohashi Y, Mizuiri S, Aikawa A, Sakai K. Association between ratio of measured extracellular volume to expected body fluid volume and renal outcomes in patients with chronic kidney disease: a retrospective single-center cohort study. BMC nephrol. 2014;15(1):189. 25435421 10.1186/1471-2369-15-189 PMC4268815

[20] Van den HAM EC, Kooman JP, Christiaans MH, Nieman FH, Van Kreel BK, Heidendal GA, et al Body Composition in Renal Transplant Patients Bioimpedance Analysis Compared to Isotope Dilution, Dual Energy X-Ray Absorptiometry, and Anthropometry. J Am Soc Nephrol. 1999;10(5):1067–79. 10232694 10.1681/ASN.V1051067

[21] Yang J, Ryu H, Han M, Kim H, Hwang YH, Chung JW, et al Comparison of volume-reductive therapies for massive polycystic liver disease in autosomal dominant polycystic kidney disease. Hepatol Res. 2016;46(2):183–91. 10.1111/hepr.12560 26190457

[22] Adequacy of dialysis and nutrition in continuous peritoneal dialysis: association with clinical outcomes. Canada-USA (CANUSA) Peritoneal Dialysis Study Group. J Am Soc Nephrol. 1996;7(2):198–207. 8785388 10.1681/ASN.V72198

[23] Kim Y, Han BG. Cohort Profile: The Korean Genome and Epidemiology Study (KoGES) Consortium. Int J Epidemiol. 2017;46(4):1350 10.1093/ije/dyx105 28938752 PMC5837323

[24] Lee JE, Jo IY, Lee SM, Kim WJ, Choi HY, Ha SK, et al Comparison of hydration and nutritional status between young and elderly hemodialysis patients through bioimpedance analysis. Clin interv Aging. 2015;10:1327–34. 10.2147/CIA.S86229 26316728 PMC4541557

[25] Han M, Park HC, Kim H, Jo HA, Huh H, Jang JY, et al Hyperuricemia and deterioration of renal function in autosomal dominant polycystic kidney disease. BMC nephrol. 2014;15(1):1. 24739095 10.1186/1471-2369-15-63 PMC4021172

[26] O’Neill WC, Robbin ML, Bae KT, Grantham JJ, Chapman AB, Guay-Woodford LM, et al Sonographic assessment of the severity and progression of autosomal dominant polycystic kidney disease: the Consortium of Renal Imaging Studies in Polycystic Kidney Disease. Am J Kidney Dis. 2005;46(6):1058–64. 10.1053/j.ajkd.2005.08.026 16310571

[27] Levey AS, Stevens LA. Estimating GFR using the CKD epidemiology collaboration (CKD-EPI) creatinine equation: more accurate GFR estimates, lower CKD prevalence estimates, and better risk predictions. Am J Kidney Dis. 2010;55:622 10.1053/j.ajkd.2010.02.337 20338463 PMC2846308

[28] Mialich MS, Sicchieri JMF, Junior AAJ. Analysis of Body Composition: A Critical Review of the Use of Bioelectrical Impedance Analysis. Int J Clin Nutr. 2014;2(1):1–10.

[29] Liu MH, Wang CH, Huang YY, et al Edema index established by a segmental multifrequency bioelectrical impedance analysis provides prognostic value in acute heart failure. J Cardiovasc Med (Hagerstown). 2012;13(5):299–306. 22367574 10.2459/JCM.0b013e328351677f

[30] Lee Y, Kwon O, Shin CS, et al Use of Bioelectrical Impedance Analysis for the Assessment of Nutritional Status in Critically Ill Patients. Clin Nutr Res. 2015;4(1):32–40. 10.7762/cnr.2015.4.1.32 25713790 PMC4337921

[31] Kyle UG, Bosaeus I, De Lorenzo AD, et al Bioelectrical impedance analysis—part II: utilization in clinical practice. Clin Nutr. 2004;23(6):1430–53. 10.1016/j.clnu.2004.09.012 15556267

[32] Beberashvili I, Azar A, Sinuani I, et al Bioimpedance phase angle predicts muscle function, quality of life and clinical outcome in maintenance hemodialysis patients. Eur J Clin Nutr. 2014;68(6):683–9. 10.1038/ejcn.2014.67 24736681

[33] Maggiore Q, Nigrelli S, Ciccarelli C, et al Nutritional and prognostic correlates of bioimpedance indexes in hemodialysis patients. Kidney Int. 1996;50(6):2103–8. 8943496 10.1038/ki.1996.535

[34] Belarmino G, Gonzalez MC, Torrinhas RS, et al Phase angle obtained by bioelectrical impedance analysis independently predicts mortality in patients with cirrhosis. World J Hepatol. 2017;9(7):401–8. 10.4254/wjh.v9.i7.401 28321276 PMC5340995

[35] Perez Camargo DA, Allende Perez SR, Rivera Franco MM, et al Phase Angle of Bioelectrical Impedance Analysis as Prognostic Factor in Palliative Care Patients at the National Cancer Institute in Mexico. Nutr Cancer. 2017;69(4):601–6. 10.1080/01635581.2017.1299880 28353355

[36] Isoyama N, Qureshi AR, Avesani CM, Lindholm B, Bàràny P, Heimbürger O, et al Comparative Associations of Muscle Mass and Muscle Strength with Mortality in Dialysis Patients. Clin J Am Soc Nephrol. 2014;9(10):1720–8. 10.2215/CJN.10261013 25074839 PMC4186520

[37] Alvarez-Lara MA, Martin-Malo A, Espinosa M, Rodriguez-Benot A, Aljama P. Blood pressure and body water distribution in chronic renal failure patients. Nephrol Dial Transplant. 2001;16 Suppl 1:94–7. 10.1093/ndt/16.suppl_1.94 11369832

[38] Chamney PW, Wabel P, Moissl UM, Müller MJ, Bosy-Westphal A, Korth O, et al A whole-body model to distinguish excess fluid from the hydration of major body tissues. Am J Clin Nutr. 2007;85(1):80–9. 10.1093/ajcn/85.1.80 17209181

[39] Guo Q, Lin J, Li J, Yi C, Mao H, Yang X, et al The effect of fluid overload on clinical outcome in southern Chinese patients undergoing continuous ambulatory peritoneal dialysis. Perit. Dial. Int. 2015;35:691–702. 10.3747/pdi.2014.00008 26152580 PMC4690624

[40] Chen LK, Liu LK, Woo J, Assantachai P, Auyeung TW, Bahyah KS, et al Sarcopenia in Asia: consensus report of the Asian Working Group for Sarcopenia. J Am Med Dir Assoc. 2014;15:95–101. 10.1016/j.jamda.2013.11.025 24461239

[41] Cederholm T, Jensen G.L., Correia M, Gonzalez MC, Fukushima R, Higashiguchi T, et al GLIM criteria for the diagnosis of malnutrition–A consensus report from the global clinical nutrition community. Clin Nutr. 2018 10.1002/jpen.1440 30181091

