# Supplementary material for: Bioelectrical impedance analysis as a nutritional assessment tool in Autosomal Dominant Polycystic Kidney Disease
Source: PLoS One. 2019 Apr 4;14(4):e0214912. doi: 10.1371/journal.pone.0214912 (PMC6449065; doi:10.1371/journal.pone.0214912)
Supplement: S1 Table — (DOCX) [file pone.0214912.s001.docx]

**S1 Table.** **Association of BIA parameters with SGA scores according to gender**

| Parameters | Male | | | | Female | | | | Total | | | | |
| --- | --- | --- | --- | --- | --- | --- | --- | --- | --- | --- | --- | --- | --- |
|  | SGA ≤5 | SGA 6 | SGA 7 | *P* for trend | SGA ≤5 | SGA 6 | SGA 7 | *P* for trend | SGA ≤5 | SGA 6 | SGA 7 | Total | *P* for trend |
| Number of patients | 12 | 26 | 112 |  | 9 | 37 | 92 |  | 21 | 63 | 204 | 288 |  |
| **Body fluid parameters** | | | | | | | | | | | | | |
| ICW/Ht (L/m) | 13.9 ± 1.6 | 14.8 ± 1.3 | 15.6 ± 1.5 | **<0.001** | 11.8 ± 1.3 | 11.8 ± 1.1 | 11.9 ± 1.0 | 0.472 | 13.0 ± 1.8 | 13.0 ± 1.9 | 13.9 ± 2.3 | 13.7 ± 2.2 | **0.003** |
| ECW/Ht (L/m) | 8.8 ± 1.2 | 9.2 ± 0.7 | 9.6 ± 0.9 | **0.003** | 7.7 ± 0.9 | 7.5 ± 0.7 | 7.5 ± 0.6 | 0.818 | 8.3 ± 1.1 | 8.2 ± 1.1 | 8.6 ± 1.3 | 8.5 ± 1.3 | **0.029** |
| TBW/Ht (L/m) | 22.6 ± 2.7 | 24.0 ± 2.0 | 25.2 ± 2.5 | **0.001** | 19.5 ± 2.1 | 19.3 ± 1.8 | 19.4 ± 1.6 | 0.726 | 21.3 ± 2.9 | 21.3 ± 3.0 | 22.6 ± 3.6 | 22.2 ± 3.4 | **0.007** |
| **Body composition parameters** | | | | | | | | | | | | | |
| FM/Ht (kg/m) | 5.3 ± 1.9 | 7.3 ± 3.7 | 8.1 ± 3.0 | **0.006** | 9.3 ± 4.3 | 10.3 ± 2.7 | 10.0 ± 3.5 | 0.774 | 7.0 ± 3.7 | 9.0 ± 3.4 | 8.9 ± 3.4 | 8.8 ± 3.4 | 0.280 |
| FFM/Ht (kg/m) | 30.8 ± 3.7 | 32.7 ± 2.8 | 34.3 ± 3.4 | **0.001** | 26.7 ± 3.0 | 26.3 ± 2.5 | 26.5 ± 2.1 | 0.736 | 29.0 ± 4.0 | 29.0 ± 4.1 | 30.8 ± 4.8 | 30.2 ± 4.7 | **0.007** |
| LM/Ht_WB_ (kg/m) | 29.0 ± 3.5 | 30.8 ± 2.6 | 32.2 ± 3.5 | **0.001** | 24.9 ± 2.7 | 24.7 ± 2.4 | 24.9 ± 2.0 | 0.662 | 27.3 ± 3.7 | 27.2 ± 3.9 | 28.9 ± 4.7 | 28.4 ± 4.5 | **0.009** |
| LM/Ht_TR_ (kg/m) | 13.0 ± 1.1 | 14.1 ± 1.1 | 14.8 ± 1.4 | **<0.001** | 11.0 ± 1.1 | 11.2 ± 1.0 | 11.3 ± 1.0 | 0.410 | 12.2 ± 1.5 | 12.4 ± 1.8 | 13.2 ± 2.1 | 12.9 ± 2.1 | **0.002** |
| LM/Ht_UE_ (kg/m) | 3.1 ± 0.4 | 3.5 ± 0.4 | 3.7 ± 0.5 | **<0.001** | 2.5 ± 0.4 | 2.5 ± 0.4 | 2.5 ± 0.4 | 0.472 | 2.9 ± 0.5 | 2.9 ± 0.6 | 3.2 ± 0.8 | 3.1 ± 0.7 | **0.003** |
| LM/Ht_LE_ (kg/m) | 9.8 ± 1.8 | 10.2 ± 1.0 | 11.0 ± 1.1 | **<0.001** | 7.9 ± 1.0 | 8.0 ± 1.1 | 8.0 ± 0.8 | 0.552 | 9.0 ± 1.8 | 8.9 ± 1.5 | 9.6 ± 1.8 | 9.4 ± 1.7 | **0.006** |

BIA; bioelectrical impedance analysis, ICW/Ht; height-adjusted intracellular water, ECW/Ht; height-adjusted extracellular water, TBW/Ht; height-adjusted total body water, FM/Ht; height-adjusted fat mass, FFM/Ht; height-adjusted fat free mass, LM/Ht_WB_; height-adjusted lean mass of whole-body, LM/Ht_TR_; height-adjusted lean mass of trunk, LM/Ht_UE_; height-adjusted lean mass of upper extremities, LM/Ht_LE_; height-adjusted lean mass of lower extremities, SGA; subjective global assessment
