# Supplementary material for: Bioelectrical impedance analysis as a nutritional assessment tool in Autosomal Dominant Polycystic Kidney Disease
Source: PLoS One. 2019 Apr 4;14(4):e0214912. doi: 10.1371/journal.pone.0214912 (PMC6449065; doi:10.1371/journal.pone.0214912)
Supplement: S2 Table — (DOCX) [file pone.0214912.s002.docx]

**S2 Table. Baseline patient characteristics according to nutritional status as evaluated by SGA in subgroup analysis among CKD stage 1-3A**

| Parameters | SGA 5 | SGA 6 | SGA 7 | Total | *P* for trend |
| --- | --- | --- | --- | --- | --- |
| Number of patients | 10 (4.5%) | 42 (19%) | 169 (76.5%) | 221 |  |
| Female | 4 (40%) | 26 (61.9%) | 80 (47.3%) | 110 (49.8%) | 0.436 |
| Age (years) | 51.5 ± 13.6 | 49.5 ± 12.4 | 45.0 ± 11.7 | 46.2 ± 12.0 | **0.014** |
| Height (cm) | 164.2 ± 4.9 | 163.5 ± 8.8 | 167.6 ± 9.9 | 166.7 ± 9.7 | **0.01** |
| Weight (kg) | 59.7 ± 6.5 | 61.8 ± 10.6 | 66.8 ± 12.5 | 65.6 ± 12.1 | **0.004** |
| BMI (kg/m^2^) | 22.2 ± 2.6 | 23.0 ± 2.6 | 23.6 ± 3.0 | 23.4 ± 2.9 | 0.09 |
| Hemoglobin (g/dL) | 12.6 ± 1.4 | 13.4 ± 1.3 | 13.8 ± 1.5 | 13.7 ± 1.5 | **0.01** |
| Serum creatinine (mg/dL) | 1.1 ± 0.3 | 1.0 ± 0.3 | 1.0 ± 0.2 | 1.0 ± 0.3 | 0.614 |
| eGFR (mL/min/1.73 m^2^) | 70.7 ± 18.5 | 70.0 ± 20.0 | 76.6 ± 19.9 | 75.1 ± 20.0 | **0.041** |
| Protein (g/dL) | 7.4 ± 0.5 | 7.3 ± 0.4 | 7.3 ± 0.4 | 7.3 ± 0.4 | 0.434 |
| Albumin (g/dL) | 4.4 ± 0.2 | 4.3 ± 0.2 | 4.4 ± 0.3 | 4.4 ± 0.3 | 0.581 |
| Total cholesterol (mg/dL) | 185.3 ± 22.2 | 176.5 ± 29.0 | 178.7 ± 26.0 | 178.6 ± 26.1 | 0.923 |
| htTKLV (mL/m) | 2,112 [1382;3710] | 1,893 [1393;2660] | 1,556 [1288;2032] | 1610 [1603;2132] | **0.015** |
| htTKV (mL/m) | 578 [420;881] | 679 [437;1160] | 568 [367;847] | 587 [377;880] | 0.163 |
| htTLV (mL/m) | 1,692 [830;2831] | 985 [819;1393] | 946 [807;1127] | 954 [812;1152] | 0.100 |
| CKD stage |  |  |  |  | **0.008** |
| 1 | 1 (10%) | 7 (16.7%) | 44 (26.0%) | 52 (23.5%) |  |
| 2 | 5 (50%) | 19 (45.2%) | 92 (54.4%) | 116 (52.5%) |  |
| 3A | 4 (40%) | 16 (38.1%) | 33 (19.5%) | 53 (24.0%) |  |

BMI; body mass index, CKD; chronic kidney disease, eGFR; estimated glomerular filtration rates, htTKLV; height-adjusted total abdominal volume, htTKV; height-adjusted total kidney volume, htTLV; height-adjusted total liver volume, SGA, subjective global assessment
