# Supplementary material for: Bioelectrical impedance analysis as a nutritional assessment tool in Autosomal Dominant Polycystic Kidney Disease
Source: PLoS One. 2019 Apr 4;14(4):e0214912. doi: 10.1371/journal.pone.0214912 (PMC6449065; doi:10.1371/journal.pone.0214912)
Supplement: S3 Table — (DOCX) [file pone.0214912.s003.docx]

**S3 Table. Association of BIA parameters with SGA scores among CKD stage 1-3A**

| Parameters | SGA 5 | SGA 6 | SGA 7 | Total | *P* for trend |
| --- | --- | --- | --- | --- | --- |
| Number of patients | 10 (4.5%) | 42 (19%) | 169 (76.5%) | 221 |  |
| **Body fluid parameters** | | | | | |
| ICW/Ht (L/m) | 13.0 ± 1.6 | 12.9 ± 2.0 | 13.8 ± 2.3 | 13.6 ± 2.3 | **0.014** |
| ECW/Ht (L/m) | 8.2 ± 0.9 | 8.1 ± 1.1 | 8.6 ± 1.3 | 8.4 ± 1.3 | **0.043** |
| TBW/Ht (L/m) | 21.3 ± 2.5 | 21.0 ± 3.0 | 22.4 ± 3.6 | 22.0 ± 3.5 | **0.019** |
| ECW/TBW_WB_ | 0.389 ± 0.009 | 0.386 ± 0.007 | 0.383 ± 0.007 | 0.384 ± 0.007 | **0.004** |
| ECW/TBW_TR_ | 0.388 ± 0.01 | 0.386 ± 0.009 | 0.382 ± 0.007 | 0.383 ± 0.008 | **0.001** |
| ECW/TBW_UE_ | 0.379 ± 0.004 | 0.378 ± 0.004 | 0.378 ± 0.004 | 0.378 ± 0.004 | 0.285 |
| ECW/TBW_LE_ | 0.393 ± 0.010 | 0.389 ± 0.008 | 0.386 ± 0.007 | 0.387 ± 0.008 | **0.003** |
| **Body composition** | | | | | |
| FM/Ht (kg/m) | 7.4 ± 4.6 | 9.1 ± 3.3 | 9.1 ± 3.4 | 9.1 ± 3.5 | 0.5 |
| FFM/Ht (kg/m) | 28.9 ± 3.4 | 28.5 ± 4.1 | 30.5 ± 4.9 | 30.1 ± 4.8 | **0.019** |
| LM/Ht_WB_(kg/m) | 27.2 ± 3.2 | 26.8 ± 3.9 | 28.6 ± 4.8 | 28.3 ± 4.6 | **0.024** |
| LM/Ht_TR_ (kg/m) | 12.2 ± 1.5 | 12.3 ± 1.9 | 13.1 ± 2.1 | 12.9 ± 2.1 | **0.011** |
| LM/Ht_UE_ (kg/m) | 2.8 ± 0.5 | 2.8 ± 0.6 | 3.1 ± 0.8 | 3.1 ± 0.8 | **0.013** |
| LM/Ht_LE_ (kg/m) | 9.1 ± 1.3 | 9.6 ± 5.9 | 9.6 ± 1.8 | 9.6 ± 3.0 | **0.026** |
| **Nutritional parameters** | | | | | |
| PhA_WB_ (θ) | 5.6 ± 0.7 | 5.3 ± 0.8 | 5.4 ± 0.7 | 5.4 ± 0.7 | 0.789 |
| PhA_TR_ (θ) | 8.5 ± 1.5 | 8.8 ± 1.4 | 8.9 ± 1.4 | 8.9 ± 1.4 | 0.113 |
| PhA_UE_ (θ) | 4.8 ± 1.0 | 5.0 ± 0.8 | 5.4 ± 0.8 | 5.3 ± 0.8 | **0.013** |
| PhA_LE_ (θ) | 4.9 ± 0.7 | 5.0 ± 0.7 | 5.3 ± 0.7 | 5.2 ± 0.7 | **0.005** |

BIA; bioelectrical impedance analysis, CKD; chronic kidney disease, ECW/TBW_WB_; ratio of extracellular water to total body water of whole-body, ECW/TBW_TR_; ratio of extracellular water to total body water of trunk, ECW/TBW_UE_; ratio of extracellular water to total body water of upper extremities, ECW/TBW_LE_; ratio of extracellular water to total body water of lower extremities, FM/Ht; height-adjusted fat mass, FFM/Ht; height-adjusted fat free mass, LM/Ht_WB_; height-adjusted lean mass of whole-body, LM/Ht_TR_; height-adjusted lean mass of trunk, LM/Ht_UE_; height-adjusted lean mass of upper extremities, LM/Ht_LE_; height-adjusted lean mass of lower extremities, PhA_WB_; Phase angle of whole-body, PhA_TR_; Phase angle of trunk, PhA_UE_ ;Phase angle of upper extremities, PhA_LE_ ; Phase angle of lower extremities, SGA; subjective global assessment
