# Supplementary figures and images for: Bioelectrical impedance analysis as a nutritional assessment tool in Autosomal Dominant Polycystic Kidney Disease
Source: PLoS One. 2019 Apr 4;14(4):e0214912. doi: 10.1371/journal.pone.0214912 (PMC6449065; doi:10.1371/journal.pone.0214912)

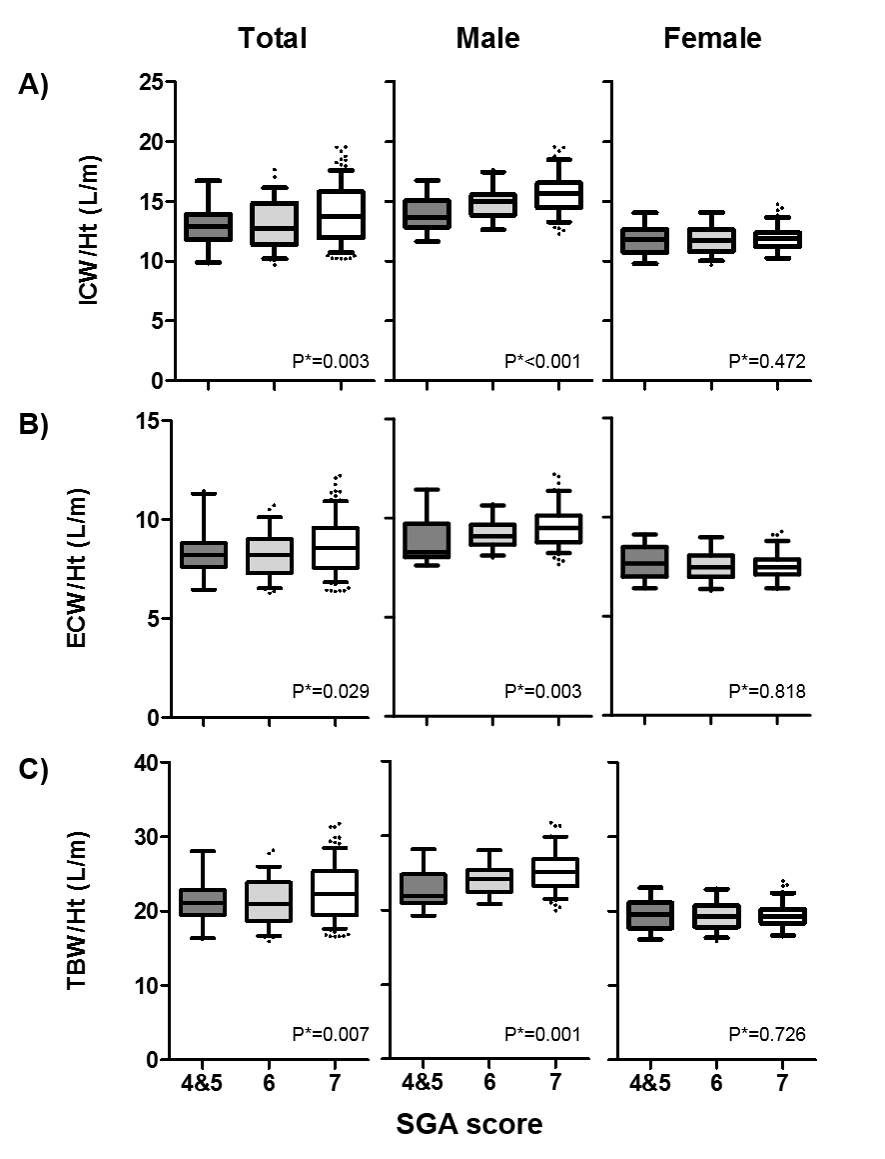

Supplement: S1 Fig — (A) ICW/Ht (L/m), (B) ECW/Ht (L/m), (C) TBW/Ht (L/m). ICW/Ht, ECW/Ht and TBW/Ht did not show significant association with SGA scores in female population. *P; P for trends SGA 7, well-nourished; SGA 6, at risk; SGA 5, mildly malnourished; SGA 3–4, moderately ICW/Ht; height-adjusted intracellular water, ECW/Ht; height-adjusted extracellular water, TBW/Ht; height-adjusted total body water, SGA; subjective global assessment. (TIF) [file pone.0214912.s004.tif]

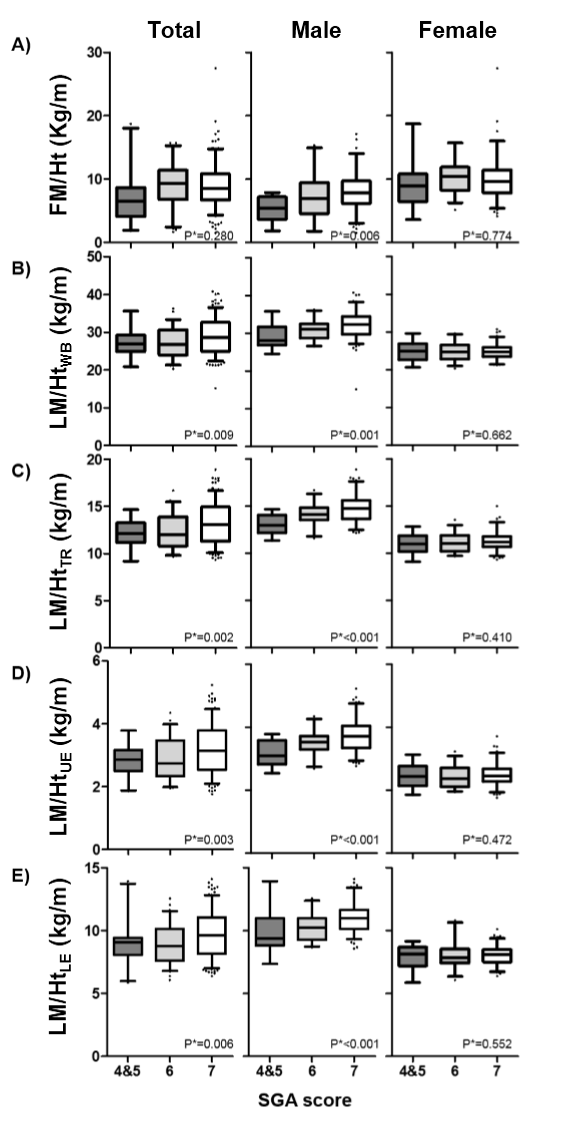

Supplement: S2 Fig — (A) FM/HtWB (kg/m), (B) LM/HtWB (kg/m), (C) LM/HtTR (kg/m), (D) LM/HtUE (kg/m), and (E) LM/HtLE (kg/m). All body composition parameter did not show significant association with SGA scores in female population. *P; P for trends SGA 7, well-nourished; SGA 6, at risk; SGA 5, mildly malnourished; SGA 3–4, moderately FM/Ht; height-adjusted fat mass, FFM/Ht; height-adjusted fat free mass, LM/HtWB; height-adjusted lean mass of whole-body, LM/HtTR; height-adjusted lean mass of trunk, LM/HtUE; height-adjusted lean mass of upper extremities, LM/HtLE; height-adjusted lean mass of lower extremities, SGA; subjective global assessment. (TIF) [file pone.0214912.s005.tif]

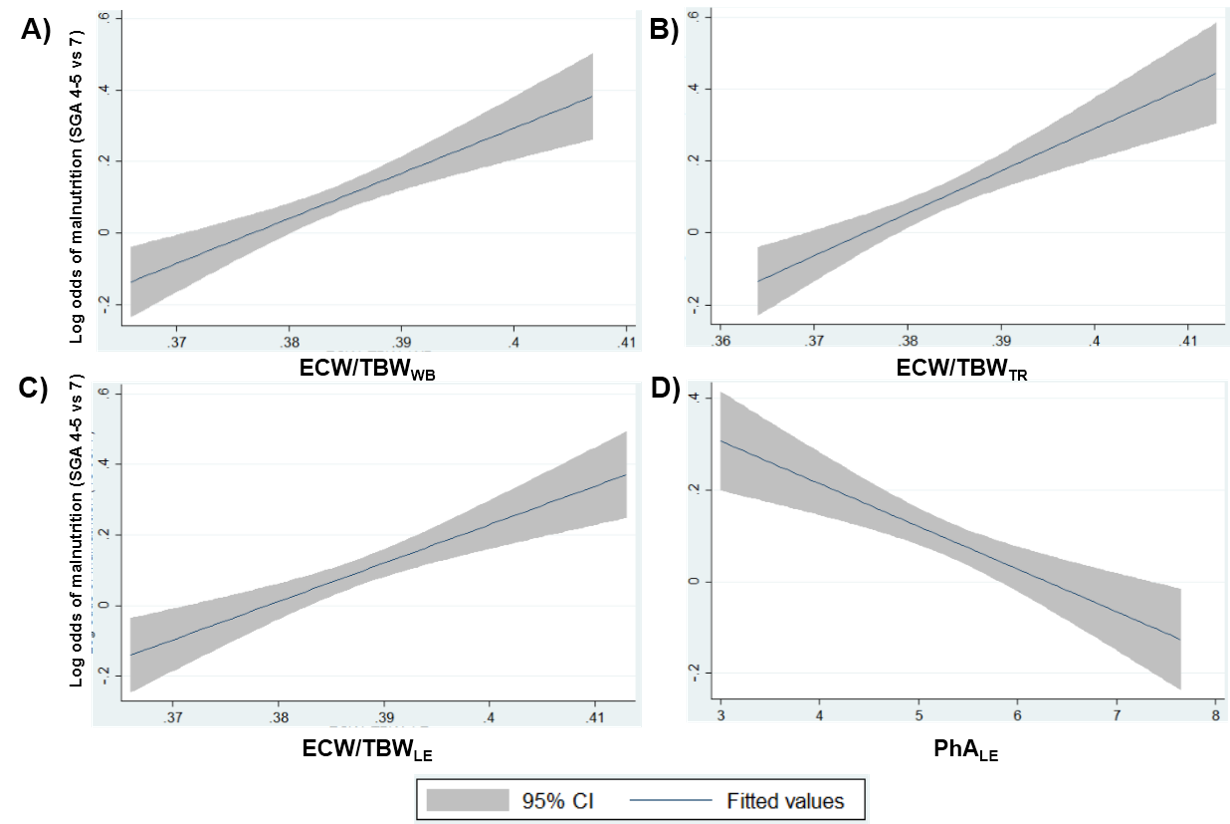

Supplement: S3 Fig — A) ECW/TBWWB, (B) ECW/TBWTR, (C) ECW/TBWLE and (D) PhALE (θ), after adjusted with age, hemoglobin and renal function In the adjusted likelihood of having malnutrition showed positive correlation with ECW/TBWWB, TR and LE and negative correlation with PhALE BIA; bioelectrical impedance analysis, ECW/TBWWB; ratio of extracellular water to total body water of whole-body, ECW/TBWTR; ratio of extracellular water to total body water of trunk, ECW/TBWLE; ratio of extracellular water to total body water of lower extremities, PhALE; Phase angle of lower extremities, SGA; subjective global assessment. (TIF) [file pone.0214912.s006.tif]

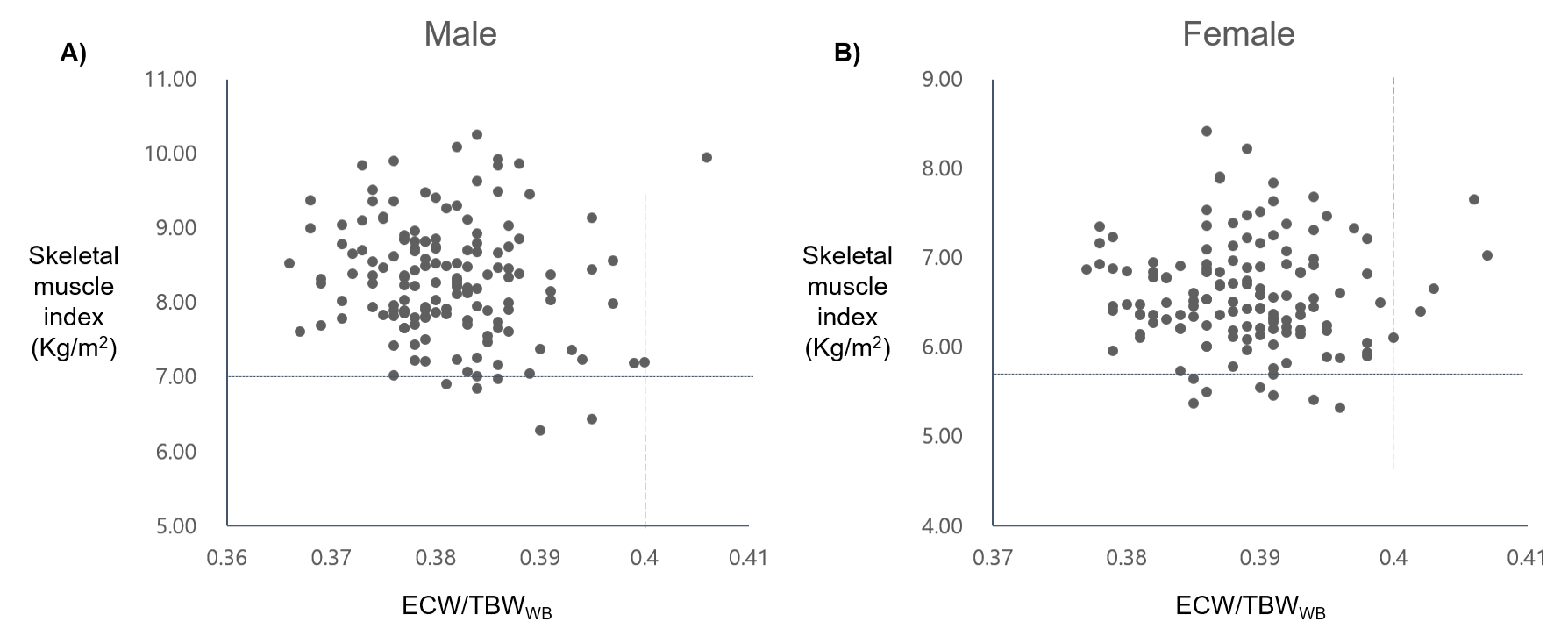

Supplement: S4 Fig — The cutoff value of ECW/TBW for edema was 0.4 and skeletal muscle index for sarcopenia was a) 7.0 kg/m2 for male and 5.7 kg/m2 for female, respectively. In the subject group with both ECW/TBWWB > 0.4 and skeletal muscle index higher than the cutoff for sarcopenia, the lean mass could be overestimated because over-hydration are included in the lean mass. However, in our study population, majority of subjects were not included in this group suggesting the minimal effect of volume overload on lean mass measured by BIA. BIA; bioelectrical impedance analysis, ECW/TBW WB; ratio of extracellular water to total body water of whole-body. (TIF) [file pone.0214912.s007.tif]
